# Nephrotoxicity caused by oral antiviral agents in patients with chronic hepatitis B treated in a hospital for tropical diseases in Thailand

**DOI:** 10.1186/s40360-015-0037-6

**Published:** 2015-12-14

**Authors:** Aung Myint Thu, Kittiyod Poovorawan, Chatporn Kittitrakul, Apichart Nontprasert, Natthida Sriboonvorakul, Weerapong Phumratanaprapin, Pisit Tangkijvanich, Wattana Leowattana, Polrat Wilairatana

**Affiliations:** Department of Clinical Tropical Medicine, Faculty of Tropical Medicine, Mahidol University, Bangkok, Thailand; Research Unit of Hepatitis and Liver Cancer, Department of Biochemistry, Faculty of Medicine, Chulalongkorn University, Bangkok, Thailand

**Keywords:** Nephrotoxicity, Antiviral agents, Nucleotide, Nucleoside, Chronic hepatitis B, Thailand

## Abstract

**Background:**

There is increasing concern about the potential for nephrotoxicity in patients with chronic hepatitis B (CHB) treated long-term with nucleotide analogs.

**Methods:**

We examined renal dysfunction and its associated risk factors in patients with CHB treated with antiviral regimens containing either nucleosides or nucleotide analogs. We undertook a retrospective cohort study from 2006 to 2014 at the Hospital for Tropical Diseases, Bangkok, Thailand, and analyzed the data of 102 patients with a median follow-up time of 44.5 months (range 4–101 months).

**Results:**

Seventy-three patients were treated with an antiviral regime containing a nucleoside analog, and 29 with a regime containing a nucleotide analog. Abnormally elevated serum creatinine concentration was observed in 12 patients (11.8 %) after 8 years of treatment. Thirty one percent of patients treated with nucleotide analogs had elevated serum creatinine levels and three of these patients (10.3 %) developed nephrotoxicity. In contrast, serum creatinine concentrations were elevated in three of the 73 patients treated with a nucleoside analog (4.1 %), and none developed nephrotoxicity. The incidence of renal dysfunction by the nucleotide analog regimen was cumulative, with 11.1, 21.0, 26.5 and 47.6 % of patients affected after 2, 4, 6 and 8 years, respectively. Univariate and multivariate analysis indicated that a nucleotide analog-based regimen significantly predicted renal dysfunction (odds ratio 10.5, 95 % confidence intervals 2.6–42.4, *P* <0.001).

**Conclusion:**

The long-term use of nucleotide analogs increased the risk of nephrotoxicity in patients with CHB. Thus, the regular assessment of renal function is recommended for all patients with CHB, particularly those treated with a nucleotide analog.

## Background

More than 350 million people are estimated to have chronic hepatitis B virus (HBV) infection. Patients may be asymptomatic, but can develop severe hepatic impairment. Approximately 15–40 % of chronic HBV cases progress to cirrhosis, end-stage liver failure or hepatocellular carcinoma [1].

Currently, five oral nucleoside or nucleotide analogs are approved for the treatment of chronic hepatitis B (CHB) by the United States Food and Drug Administration: lamivudine (LAM), entecavir (ETV), telbivudine (LdT), adefovir (ADV) and tenofovir (TDF). Currently, all of these antiviral drugs are approved by the Thai FDA for the treatment of CHB. Nucleoside and nucleotide analogs target HBV DNA polymerase protein, a multifunctional protein essential for viral replication [2].

LAM is a nucleoside analog and was the first oral agent approved for the treatment of CHB [3]. Although LAM resistance has emerged, LAM appears not to impair renal function [4]. ETV has potent antiviral activity with low rates of drug resistance, but the establishment of LAM resistance diminishes its efficacy because genotypic resistance to ETV develops more frequently in prior LAM-treated patients [5]. In clinical trials, ETV had a similar safety and tolerability profile to LAM [6], with only minor adverse events reported and no evidence of mitochondrial or other serious renal side effects in patients treated for up to 5 years. LdT is an L-nucleoside with potent activity against HBV, but its role in the treatment of CHB as monotherapy is limited by cross-resistance with LAM [3]. Generally LdT is well tolerated, and its safety profile appeared to be similar to LAM in registration trials.

Resistance to LAM is an increasing problem in the treatment of CHB [2]. Given the potential for cross-resistance to LdT and ETV in patients refractory to LAM, treatment with nucleotide analogs is preferred. Treatment with oral antiviral agents might require life-long administration. Clinical trials of ADV and TDF also concluded that they have an acceptable safety profile, but following their widespread use in clinical practice there have been reports that the long-term use of nucleotide analogs may impair renal function [2].

In one large randomized clinical trial, ADV exhibited dose-dependent nephrotoxicity [7]; however, no significant rise in serum creatinine concentration was observed in patients taking ADV compared with placebo in three other randomized controlled trials [8–10]. Ultimately ADV was given regulatory approval at a daily dose of 10 mg. Nucleotide analog-related nephrotoxicity has been reported in subsequent cohort studies, with a prevalence of 3.0–9.9 % for ADV monotherapy or ADV-LAM combination therapy [4, 11–13].

According to updated guidelines, TDF can also be used as a first-line treatment option for CHB [3]. A trial comparing TDF with ADV found that a 300 mg daily dose of TDF had superior antiviral efficacy and a good safety profile, with no adverse renal events reported during the 48-week follow-up period [14]. The nephrotoxicity of TDF was already observed for the treatment of human immunodeficiency virus (HIV), but in HIV other antiviral drugs might also contribute to renal dysfunction. One recent retrospective study found that 5.6 % of patients with CHB had evidence of nephrotoxicity after 2 years of treatment with TDF [15]. In a recently published 7-year safety and efficacy study of TDF in CHB, 3.6 % of patients had developed nephrotoxicity [16].

Renal toxicity may also be manifested by proximal tubular cell toxicity without a rise in serum creatinine concentration, including a Fanconi-like syndrome characterized by proteinuria, normoglycemic glycosuria and hyperphosphaturia [17, 18]. Several cases of this syndrome have been reported in patients taking nucleotide analogs for CHB [19, 20]. Differences in the age, body weight, baseline renal function, prevalence of hypertension, diabetes mellitus and cirrhosis in previous clinical trials and cohorts may also account for the differences in renal dysfunction reported after treatment with nucleotide analogs [11, 13, 19]. Importantly, renal dysfunction caused by nucleotide analogs appears to be reversible, and if renal impairment is detected promptly any further deterioration can be avoided with the correct intervention.

This study examined the long-term renal safety of currently approved nucleoside and nucleotide analogs, and determined the risk factors for renal dysfunction in patients with CHB treated in a hospital for tropical diseases in Thailand.

## Methods

We undertook a retrospective observational study of patients with CHB who regularly attended the hepatology outpatient clinic at the Hospital for Tropical Diseases, Bangkok, Thailand between 2006 and 2014. Patient information was anonymized and de-identified prior to analysis.

We included all patients diagnosed with CHB on the basis of a positive test for serum HBV surface antigen (HBsAg) for more than 6 months who had been treated with any oral antiviral agent. We excluded patients who had hepatitis C virus or HIV co-infection, patients with advanced kidney disease (such as those requiring dialysis or renal transplant recipients) and patients treated with other drugs that have impacts on renal function.

### Evaluation of renal dysfunction

Serum creatinine concentration was categorized according to the National Institute of Allergy and Infectious Diseases Common Toxicity Grading Scale [9]. Grade 1 is defined as ≥0.5 mg/dl above baseline, Grade 2 as 2.1–3.0 mg/dl, Grade 3 as 3.1–6.0 mg/dl and Grade 4 as >6.0 mg/dl. Renal abnormality was also assessed by urinalysis for glycosuria and proteinuria. An abnormal rise in serum creatinine (ARSC) or renal dysfunction is defined as an increase ≥0.3 mg/dl from baseline or a 1.5-fold increase from baseline.

### Comorbidities

Arterial hypertension was defined as a history of hypertensive disease, treatment with antihypertensive drugs or a resting systolic blood pressure ≥140 mmHg and/or a diastolic blood pressure ≥90 mmHg [21]. Diabetes mellitus was defined as a fasting plasma glucose concentration >126 mg/dl on two consecutive occasions, or treatment with oral hypoglycemic agents or insulin. Liver cirrhosis was identified by clinical and ultrasound examination.

### Data collection

The records of all patients with CHB were reviewed from the time that antiviral treatment was started. Patients were identified by their attendance at the outpatient hepatology clinic and by searches of the hospital registry, in which diagnosis is recorded according to the International Classification of Diseases. After screening against the inclusion and exclusion criteria, 159 patients were eligible for the study. Of these, patients in whom there had not been regular long-term follow-up of serum creatinine concentration were excluded. In total, the data of 102 patients were analyzed in the study. Patients were divided into two groups: those treated with a nucleoside analog (NSA) and those treated with a nucleotide analog (either alone or in combination therapy with a nucleoside analog, NTA).

### Statistical analysis

SPSS version 18 software (SPSS Inc., Chicago, IL) was used for all statistical analyses. Demographic data and the results of baseline laboratory investigations were summarized using descriptive techniques for continuous and categorical data. The Student’s *t*-test was used to compare baseline and post-treatment serum creatinine concentration in each treatment group. Fisher’s and the chi-squared tests were used to establish the nature of relationships between antiviral drug therapy and renal dysfunction, and other risk factors for renal dysfunction. Kaplan-Meier survival analysis and the log-rank test were undertaken to compare the cumulative renal outcomes of each group. Cox proportional hazards regression analysis was performed to determine independent risk factors for renal dysfunction. A *P* value < 0.05 was considered statistically significant.

### Ethics approval

The study was conducted with the approval of the Hospital for Tropical Diseases, Bangkok, Thailand. The protocol was approved by the Institutional Review Board of the Faculty of Tropical Medicine, Mahidol University, Thailand (MUTM 2014-076-01).

## Results

### Baseline characteristics of the study population

**Fig. 1:**
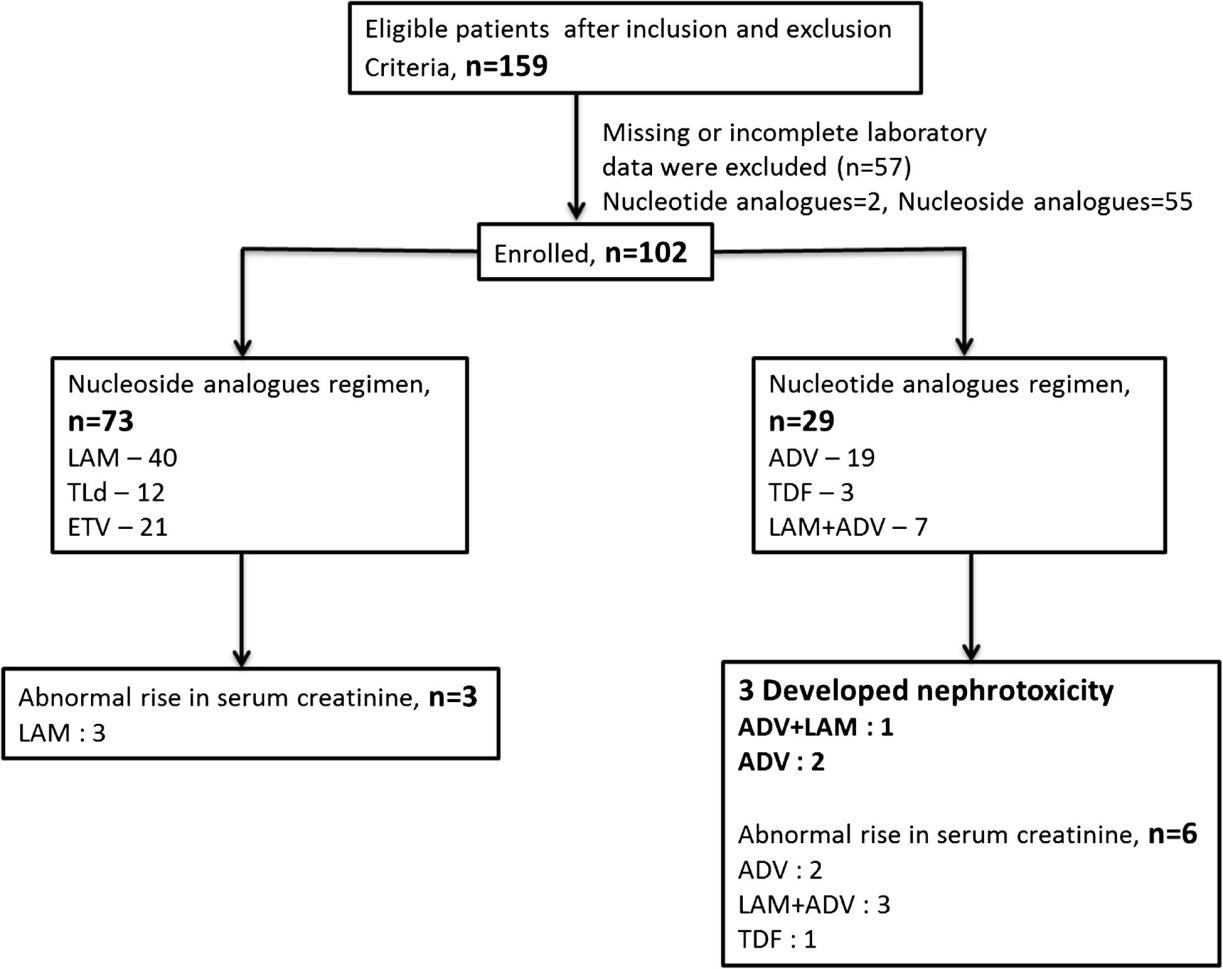
Study flowchart. LAM, lamivudine; ETV, entecavir; LdT, telbivudine; ADV, adefovir; TDF, tenofovir

Of the CHB patients who were screened, 159 were eligible for the study; however, 57 patients without regular long-term serum creatinine data were excluded and consequently the data of 102 patients were subject to analysis. Of these, 73 were treated with nucleoside analogs (40 with LAM, 12 with TLd and 21 with ETV) and were allocated to the NSA group. Twenty-nine patients were treated with nucleotide analogs either as monotherapy or in combination with a nucleoside analog (19 were treated with ADV alone, three with TDF alone and seven with LAM + ADV; Fig. 1), and were allocated to the NTA group. All the baseline characteristics data were obtained before the initiation of treatment in all cases.

The majority of patients treated with nucleoside and nucleotide analogs were male (67.1 and 82.7 %, respectively). The NTA group was significantly older than the NSA group (mean 56.6 years ± standard deviation 6.2 years *versus* 52.1 years ± 11.3 years, *P* = 0.011) and the proportion with hypertension was significantly higher (37.9 % *versus* 17.8 %, *P* = 0.031). There was no significant difference in the proportion of patients positive for hepatitis B e antigen (HBeAg), baseline serum alanine transaminase, HBV DNA, serum creatinine concentrations, or mean duration of treatment between the NSA and NTA groups (Table 1).

**Table 1:** Demographic and clinical characteristics of the study cohort

Characteristic at baseline	Nucleoside analog regimen (*n* = 73)	Nucleotide analog regimen (*n* = 29)	*P* value
Age (years)	52.1 ± 11.3	56.6 ± 6.2	0.011*
Male sex (%)	49 (67.1 %)	24 (82.7 %)	0.114
Weight (kg)	67.8 ± 14.4	66.5 ± 14.4	0.645
Diabetes mellitus (%)	9 (12.3 %)	4 (13.8 %)	0.841
Hypertension (%)	13 (17.8 %)	11 (37.9 %)	0.031*
Cirrhosis (%)	4 (5.5 %)	3 (10.3 %)	0.381
Duration of treatment (months)	44.5 (3–105)	52.0 (3–103)	0.643
HBV e antigen positive (%)	21 (28.8 %)	11 (37.9 %)	0.364
Serum ALT concentration (U/l)	56 (15–479)	33 (13–340)	0.125
HBV DNA concentration (log IU/ml)	4.99 ± 1.73	4.86 ± 1.40	0.735
Serum creatinine concentration (mg/dl)	0.87 ± 0.18	0.84 ± 0.17	0.417

Data are presented as the mean ± standard deviation, the median (range) or the number (proportion, %). *P <0.05

*HBV* hepatitis B virus, *ALT* alanine transaminase

**Fig. 2:**
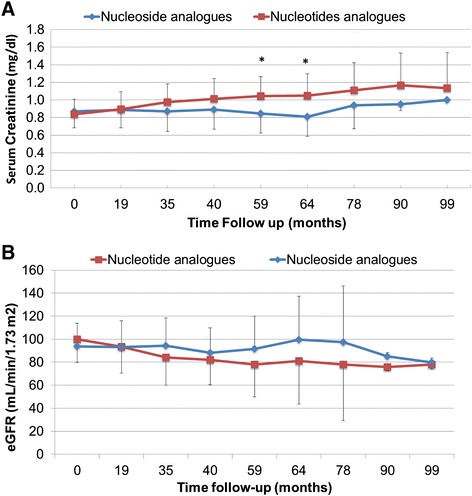
Mean serum creatinine concentration (**a**) and eGFR (**b**) in the nucleoside and nucleotide analog-treated groups over a 96-month follow-up period. *P < 0.05

There was no significant difference in the baseline serum creatinine concentrations of the NSA and NTA groups (0.87 ± 0.18 mg/dl *versus* 0.84 ± 0.17 mg/dl, respectively; *P* = 0.417). After 19 months of treatment, the mean creatinine concentration had increased gradually in both groups, but was significantly higher in the NTA group (from 0.84 mg/dl at baseline [range 0.5–1.1 mg/dl] to 0.93 mg/dl [range 0.6–1.7 mg/dl]) at the end of the follow up period (*P* < 0.001, Fig. 2a). eGFR was also significantly reduced from baseline in the NTA group after 19 months of treatment (Fig. 2b).

### Nephrotoxicity and renal dysfunction

Nephrotoxicity was observed in three of the 29 patients in the NTA group (10.3 %): two were taking ADV and one ADV + LAM. Six of 29 (20.7 %) patients exhibited ARSC: two were taking ADV, three LAM + ADV and one TDF. There were no incidences of nephrotoxicity in the NSA group, and only three of 73 patients (4.1 %) had ARSC in the follow-up period.

**Fig. 3:**
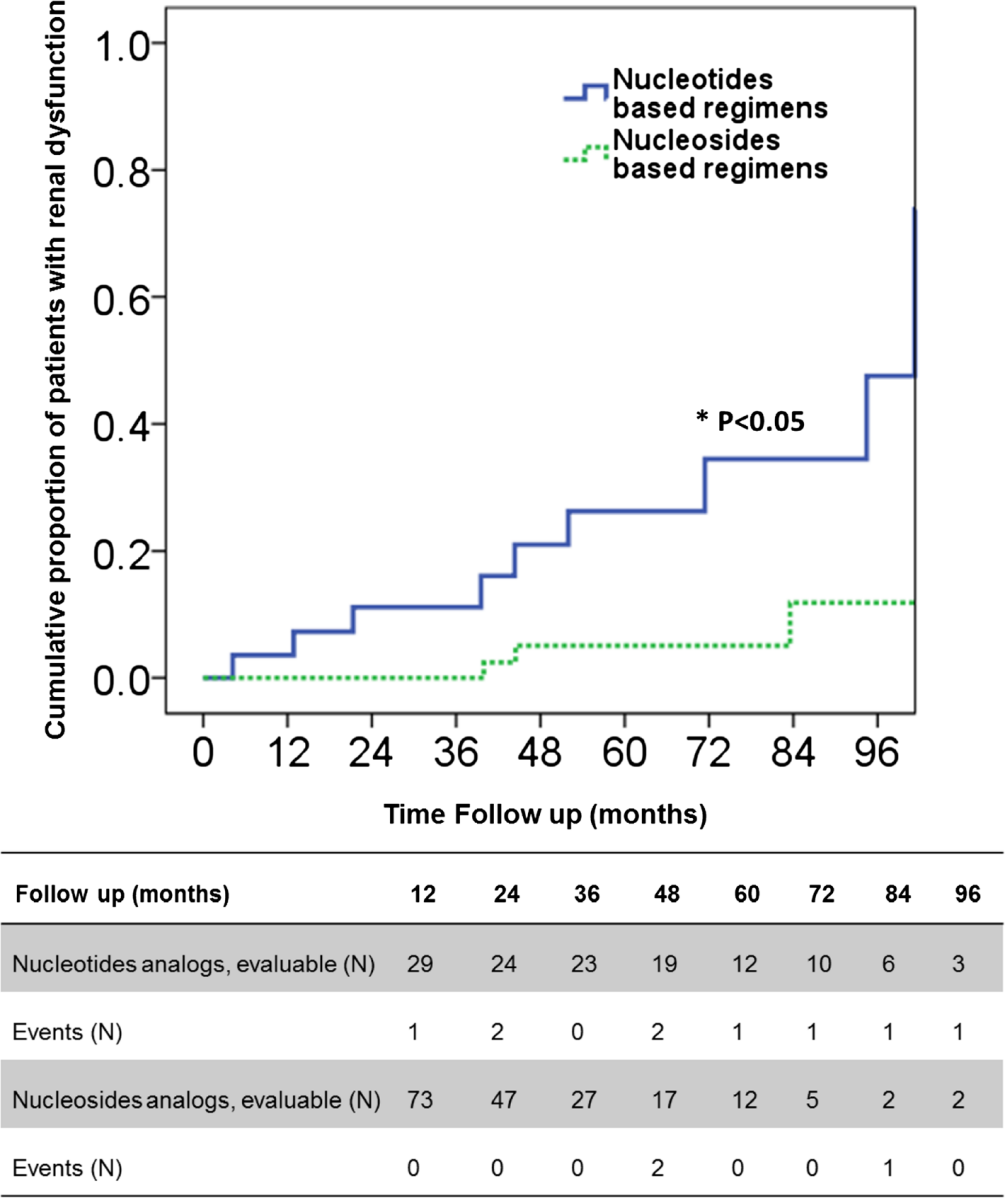
Proportion of patients developing oral antiviral-related renal dysfunction during the 96-month follow-up period. *Statistical significant by Log-rank test

The proportion of patients that developed renal dysfunction in the NTA group was significantly greater than in the NSA group (log rank test *P* < 0.001). The cumulative proportions that developed renal dysfunction in the NTA group at year 2, 4, 6 and 8 were 11.1, 21.0, 26.5 and 47.6 %, respectively. The median time to onset of renal dysfunction was 44.5 months (range 4–101 months). In contrast, only 11.9 % of the NSA group had renal dysfunction after 8 years of treatment (Fig. 3).

Urinalysis results were only available for 42 of 102 patients (41.2 %), and 19 of these 42 patients who had urinalysis results were treated with NTA. Neither glycosuria nor proteinuria was documented before or during the treatment period in these patients.

### Risk factors for renal dysfunction

Univariate and multivariate analyses were performed to establish the risk factors for renal dysfunction. A therapeutic regime containing a nucleotide analog was an independent risk factor for renal dysfunction (odds ratio 10.5, 95 % confidence interval [CI] 2.6–42.4, *P* < 0.001). Other factors such as age ≥50 years, low body weight (<60 kg), sex, diabetes mellitus, hypertension and cirrhosis were not significantly associated with renal dysfunction in either univariate or multivariate analyses (Table 2). Further subgroup regression analysis of the NTA group showed that age ≥50 years was a marginally significant risk factor for the deterioration of renal function (hazard ratio 11.14, 95 % CI 1.02–121.65 *P* = 0.048).

**Table 2:** Predictors of renal dysfunction after treatment with oral antiviral drugs

	Univariate analysis		Multivariate analysis	
Patient characteristic, *n* = 102	Crude OR (95 % CI)	*P*	Adjusted OR (95 % CI)	*P*
Age ≥50 years	2.63 (0.54–12.74)	0.328	1.33 (0.23–7.84)	0.752
Low body weight (<60 kg)	0.58 (0.12–2.86)	0.725	1.43 (0.25–8.35)	0.689
Nucleotides (versus nucleosides)	10.50 (2.60– 42.43)	0.001*	7.36 (1.54–35.27)	0.012*
Sex (M:F)	2.14 (0.44–10.44)	0.501	1.24 (0.20–7.56)	0.816
Diabetes mellitus	1.44 (0.28–7.43)	0.649	1.53 (0.29–7.87)	0.612
Hypertension	2.67 (0.76–9.36)	0.147	2.12 (0.55–8.17)	0.274
Cirrhosis	1.27 (0.14–11.58)	1.000	9.76 (0.71–134.0)	0.088

*P <0.05

*OR* odds ratio, *CI* confidence intervals, *M* male, *F* female

Three patients who developed nephrotoxicity were on a nucleotide analog-based regime (two were taking ADV, and one LAM + ADV), all were older than 50 years and one had a clinical history of diabetes mellitus and hypertension. The maximum serum creatinine concentration was 1.7 mg/dl, and the maximum increase from baseline was 0.6 mg/dl. The median onset to nephrotoxicity was 71 months (range, 44–101 months). None of the three patients had glycosuria or proteinuria (Table 3). A significant deterioration in estimated glomerular filtration rate (eGFR) was also seen in all three patients with nephrotoxicity.

**Table 3:** Clinical features of the three patients who developed nephrotoxicity

Baseline characteristics before treatment			
N	1	2	3
Age (years)	68	54	66
Sex	M	F	M
Weight (kg)	79	54	60
Hypertension	Yes	No	No
Diabetes mellitus	Yes	No	No
Cirrhotic	No	No	No
Serum creatinine concentration (mg/dl)	1.1	0.8	1.1
Treatment	LAM + ADV	ADV	ADV
Treatment duration (months)	71	44	101
Results at the diagnosis of nephrotoxicity			
Serum creatinine concentration (mg/dl)	1.7	1.4	1.6
Proteinuria	No	No	No
Glucosuria	No	No	No
Action			
Switch/Add/Continue	ADV^a^	ADV^b^	ADV^b^

*N* patient number, *Cr* serum creatinine concentration, *LAM* lamivudine, *ADV* adefovir

^a^Change; ^b^Adjusted dosage interval to 10 mg every 48 hours according to eGFR

Nine patients developed ARSC, six from the NTA group (three were taking LAM + ADV, two ADV alone and one TDF alone) and three from the NSA group (all of whom were taking LAM). The earliest onset of ARSC was in a patient taking TDF and was observed at 4 months after it had been initiated, but this patient also had hypertension, diabetes mellitus and cirrhosis before treatment, any or all of which could have contributed to or predisposed to renal impairment. In contrast, the latest onset of ARSC was at 101 months in a patient treated with ADV. Overall, the median onset to renal dysfunction was 44.4 months (range 4–101 months).

## Discussion

In our study, 10.3 % of patients in the NTA group developed nephrotoxicity on the basis of a change in serum creatinine concentration (≥0.5 mg/dl above baseline) over 8 years. In contrast, none of the patients treated with NSA developed nephrotoxicity. However, many large-scale clinical trials concluded that there was no association between ADV (an NTA) therapy and elevated serum creatinine concentration after 48 weeks of treatment [7]. Similarly Lim and colleagues reported no significant nephrotoxicity after 48 weeks of treatment with ADV [10]. Two other clinical studies also did not report an increased incidence of renal dysfunction in patients treated with ADV compared with those who received placebo [4, 8].

The safety profile of TDF, another NTA, has mostly been established in patients with HIV infection, as TDF is one of the main components of anti-retroviral therapy. There are few safety data from the mono-infected CHB population. Tenofovir is reportedly nephrotoxic in some patients with HIV, but some of the other antiviral drugs used to treat HIV infection might also cause renal side effects. In a clinical trial of TDF in CHB, an increase in serum creatinine concentration of ≥0.5 mg/dl was observed in less than 1 % of participants after 36 months follow-up [22].

Our result supports that of Hartono and colleagues, who found that 9.9 % of those receiving ADV monotherapy developed nephrotoxicity after 7 years of treatment, although in contrast with our findings, they also reported that increasing age was a risk factor for nephrotoxicity [11]. Lampertico *et al*. reported a relatively lower incidence of nephrotoxicity of 7 % in patients treated with LAM + ADV for 45.5 months [12] and Marcellin *et al*. reported an incidence of 8 % after 5 years of ADV monotherapy [23]. Therefore, the risk of nephrotoxicity appears to increase with treatment duration.

The cumulative proportion of renal dysfunction in patients treated with nucleotide analogs in our study was 33.5 % at year 6, almost identical to the findings of another recent study conducted in an Asian population which was 33.8 % at year 6 [11, 13, 19]. We found that the proportion of patients exhibiting renal dysfunction increased to 47.6 % after 8 years. The development of oral antiviral-related renal dysfunction can occur as early as 4 months and up to 8 years or greater of treatment. The risk of renal dysfunction appears to be associated with treatment duration.

The incongruity between the incidence of nephrotoxicity reported by clinical trials and later observational studies could be explained by the short observation period, and strict inclusion and exclusion criteria, of randomized studies. In routine clinical practice, patients with risk factors that might contribute to renal dysfunction still require treatment, but these patients might have been excluded from clinical trials. Our findings support those of post-marketing surveillance and longer-term clinical observational studies, which reported nucleotide analogs are nephrotoxic [11, 12, 16]. The incidence of nephrotoxicity in our study was higher than in other studies, which may be a consequence of differences in the baseline characteristics of the study populations. Different outcome measures, definitions of renal impairment and measurement techniques make it difficult to perform direct comparisons between studies. Furthermore, our study was retrospective and there was no standardized patient management protocol.

Only one patient in our cohort had been treated with TDF. This patient developed ARSC after 4 months of TDF monotherapy, but already had a history of hypertension, diabetes mellitus, and cirrhosis before treatment. In this case we were unable to draw any conclusions about the cause of renal dysfunction. Nevertheless, two studies have reported that increasing age, low body weight, hypertension, diabetes and baseline serum creatinine concentration are predictors of renal dysfunction in patients with CHB or HIV taking ADV or TDF [24, 25].

Fanconi syndrome is increasingly recognized as a complication of long-term treatment with nucleotide analogs. It is characterized by normoglycemic glucosuria, proteinuria and phosphaturia [18]. In our study, urinalysis results were not included in the selection criteria, as they were not routinely or regularly undertaken in patients with CHB at the time our study was conducted. Thus, signs of renal tubular toxicity—including proteinuria or glycosuria—might have been overlooked.

## Conclusions

In summary, we found that the long-term exposure to nucleotide analogs, especially ADV, increased the risk of nephrotoxicity in CHB patients. The incidence of nephrotoxicity was 10.3 % after 8 years of treatment, which was higher than previous reports (3.0–9.9 %) from studies conducted over 3–7 years.

**Fig. 4:**
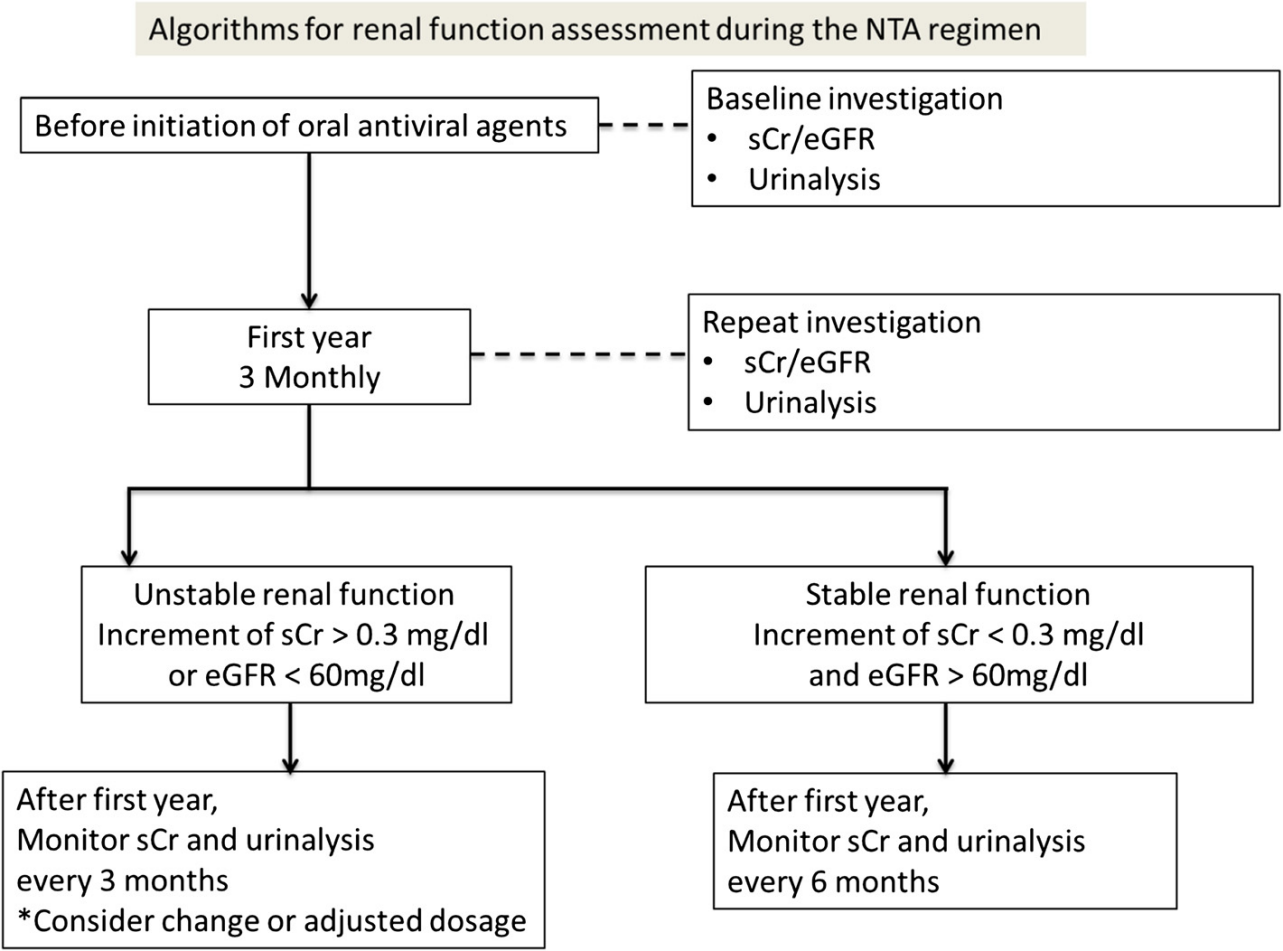
Algorithms for renal function assessment during the NTA regimen in CHB patients. eGFR, estimated glomerular filtration rate; sCr, serum creatine; NTA, nucleotide analogs

The early detection of nephrotoxicity is critical, as it is reversible and thus further deterioration of renal function can be avoided. We recommend that individuals with CHB who are taking nucleotide analogs have their renal function monitored at least every 6 months (Fig. 4), especially those with abnormal baseline results, based on the risk of deterioration of renal function and recommendations from the literature [26].

There is no unified standard for the evaluation of renal side effects in long-term nucleotide analog treatment, which may explain the inconsistent results in the literature. Therefore, well-designed, standardized long-term clinical studies are still required to determine the true incidence of renal dysfunction and establish the risk factors in patients with CHB treated with these drugs.

## Abbreviations

HBV: Hepatitis B virus
CHB: Chronic hepatitis B
HBsAg: Hepatitis B surface antigen
HBeAg: Hepatitis B e antigen
LAM: Lamivudine
ETV: Entecavir
LdT: Telbivudine
ADV: Adefovir
TDF: Tenofovir
NTA: Nucleotide analogs
NSA: Nucleoside analogs
NSAIDs: Non-steroidal anti-inflammatory drugs
HIV: Human immunodeficiency virus
